# Effect of Start-Up Strategies and Electrode Materials on Carbon Dioxide Reduction on Biocathodes

**DOI:** 10.1128/AEM.02242-17

**Published:** 2018-01-31

**Authors:** Soroush Saheb-Alam, Abhijeet Singh, Malte Hermansson, Frank Persson, Anna Schnürer, Britt-Marie Wilén, Oskar Modin

**Affiliations:** a Chalmers University of Technology, Department of Architecture and Civil Engineering, Division of Water Environment Technology, Gothenburg, Sweden; b Swedish University of Agricultural Sciences, Department of Molecular Sciences, BioGas Group, Unit of Microbiology, Uppsala, Sweden; c University of Gothenburg, Chemistry and Molecular Biology, Gothenburg, Sweden; University of Bayreuth

**Keywords:** acetogens, biocathode, cyclic voltammetry, methanogens, microbial community structure, microbial electrolysis cells, start-up strategies

## Abstract

The enrichment of CO_2_-reducing microbial biocathodes is challenging. Previous research has shown that a promising approach could be to first enrich bioanodes and then lower the potential so the electrodes are converted into biocathodes. However, the effect of such a transition on the microbial community on the electrode has not been studied. The goal of this study was thus to compare the start-up of biocathodes from preenriched anodes with direct start-up from bare electrodes and to investigate changes in microbial community composition. The effect of three electrode materials on the long-term performance of the biocathodes was also investigated. In this study, preenrichment of acetate-oxidizing bioanodes did not facilitate the start-up of biocathodes. It took about 170 days for the preenriched electrodes to generate substantial cathodic current, compared to 83 days for the bare electrodes. Graphite foil and carbon felt cathodes produced higher current at the beginning of the experiment than did graphite rods. However, all electrodes produced similar current densities at the end of the over 1-year-long study (2.5 A/m^2^). Methane was the only product detected during operation of the biocathodes. Acetate was the only product detected after inhibition of the methanogens. Microbial community analysis showed that Geobacter sp. dominated the bioanodes. On the biocathodes, the Geobacter sp. was succeeded by Methanobacterium spp., which made up more than 80% of the population. After inhibition of the methanogens, Acetobacterium sp. became dominant on the electrodes (40% relative abundance). The results suggested that bioelectrochemically generated H_2_ acted as an electron donor for CO_2_ reduction.

**IMPORTANCE** In microbial electrochemical systems, living microorganisms function as catalysts for reactions on the anode and/or the cathode. There is a variety of potential applications, ranging from wastewater treatment and biogas generation to production of chemicals. Systems with biocathodes could be used to reduce CO_2_ to methane, acetate, or other high-value chemicals. The technique can be used to convert solar energy to chemicals. However, enriching biocathodes that are capable of CO_2_ reduction is more difficult and less studied than enriching bioanodes. The effect of different start-up strategies and electrode materials on the microbial communities that are enriched on biocathodes has not been studied. The purpose of this study was to investigate two different start-up strategies and three different electrode materials for start-up and long-term operation of biocathodes capable of reducing CO_2_ to valuable biochemicals.

## INTRODUCTION

Increasing demand for fossil fuels and limited resources drive a search for renewable energy sources and environmentally friendly production methods for chemicals. Bioelectrochemical systems (BESs) constitute a new set of technologies, which has developed quickly in research laboratories worldwide during the last decade. BESs include, e.g., new methods for the treatment of wastewater, recovery of resources and energy, storage of renewable electricity as chemical fuels, and environmental sensing (1–3). Microbial fuel cells (MFCs) and microbial electrolysis cells (MECs) are two types of BESs. In MFCs, microorganisms generate electricity by oxidizing organic compounds and delivering electrons to the anode, from which the electrons travel through an external circuit to the (usually aerobic) cathode (4). MECs are typically used to generate a chemical product at the cathode. The electrons are given an extra energy boost by applying an external input voltage. In MECs, the bioanode can provide some of the reducing power needed for producing valuable chemicals, such as hydrogen gas, at the cathode (5, 6).

In BESs, living microorganisms can also serve as catalysts on the cathode. For example, biocathodes have been used to produce hydrogen (7–9), reduce carbon dioxide to acetate (10, 11) and methane (12, 13), and reduce acetate to caproate (14). Biocathodes may be especially beneficial for multielectron-reduction reactions, which are difficult or impossible to achieve on abiotic cathodes. The use of biocathodes to produce multicarbon chemicals from simple substrates is called microbial electrosynthesis (11). The concept is attractive, as it would allow environmentally friendly production of fuels and chemicals using only renewable electricity and carbon dioxide as input. However, it is more difficult to enrich biocathodes than to enrich bioanodes (8, 15). Since bioanodes need less time for start-up than biocathodes, it would be beneficial if it were possible to develop microorganisms on a bioanode and then switch them to operate as a biocathode. Rozendal et al. (7) demonstrated this concept by enriching hydrogenotrophic microorganisms on an anode surface and then switching the potential to change the electrode into a hydrogen-producing biocathode. Geelhoed and Stams (9) also showed that Geobacter sulfurreducens, known for its ability to transfer electrons to an anode, could catalyze H_2_ production when the cathode potential was lowered to about −0.6 to −0.8 V versus the standard hydrogen electrode (SHE). Pisciotta et al. (15) investigated the possibility of adapting mixed-culture anodic biofilms to cathodic conditions at different cathode potentials. Their biocathode produced hydrogen and methane, and they suggested that the technique of switching a bioanode to a biocathode can be helpful for enriching biocathodes capable of producing biofuels from carbon dioxide. Hartline and Call (16) compared different organic substrates and anode enrichment potentials for the conversion of bioanodes to biocathodes. Electrodes that had been preenriched on formate at a high anode potential (+0.15 V versus SHE) generated higher cathodic current than electrodes preenriched on acetate or at lower potential (−0.15 V versus SHE). The technique of switching bioanodes to biocathodes for the start-up of MECs looks promising. However, previous studies have not investigated the effects a switch from anodic to cathodic conditions have on an electrode's microbial community. The first goal of this study was to investigate if the microorganisms enriched on a bioanode also dominate when the electrode is switched into a biocathode or if a new microbial community develops, as well as to compare the microbial communities on biocathodes preenriched as bioanodes and on biocathodes started without preenrichment.

Moving to the biofilm level, the interface between the electrode material and the microorganisms in BESs can affect the performance and efficiency of biocathodes. Recently, it was shown that surface topography and chemistry impact the interaction, such as direct/indirect electron transfer between electrodes and microorganisms, and it was suggested that composite materials that combine high conductivity with good biocompatibility, for instance, metallic backbone with a carbon coating, lead to higher production efficiency (17). Several different electrode materials were investigated by Mohanakrishna et al. (18) in a single-chamber MEC. They suggested that VITO-CoRE (cold-rolling polymer-bound electrode) was effective as a biocathode for acetate production. The production of acetate has also been studied by Zhang et al. (19) using different modified carbon cloths and Sporomusa ovata as the sole microorganism in the cathode chamber. They suggested that carbon cloth modified by cyanuric chloride increased acetate production up to 7-fold compared to untreated carbon cloth. Carbon has been used as biocathode material in different studies, since it is inexpensive and biocompatible. Most of the previous research investigated different types of carbon electrodes during relatively short time spans. However, there is a lack of knowledge about the long-term performance of different types of carbon biocathodes. The other goal of this study was therefore to compare three types of carbon electrodes (carbon felt, graphite foil, and graphite rods) during long-term operation of biocathodes.

In the experiment, two microbial electrolysis cells (MEC1 and MEC2) were operated for over 1 year. Each reactor contained duplicate electrodes of the three tested carbon materials. Two start-up strategies were tested for the enrichment of CO_2_-reducing biocathodes. In MEC1, the electrodes were preenriched as acetate-oxidizing bioanodes by controlling the electrode potential at −0.2 V versus SHE. Then, the potential was lowered to −0.65 V versus SHE to convert the electrodes into biocathodes. In MEC2, the electrodes were operated as biocathodes at −0.65 V versus SHE from the start of the experiment. To inhibit methanogens, 2-bromoethanesulfonate was added to the reactors 1 month before the end of the experiment. This allowed us to investigate the transition from methanogenic to acetogenic biocathodes. The microbial community composition of the preenriched bioanodes and biocathodes was investigated using high-throughput sequencing of the V4 region of the 16S rRNA gene (20). Acetogens were expected to play a key role on the biocathodes (especially after the inhibition of methanogens). Therefore, community changes were also assessed using terminal restriction fragment length polymorphism (TRFLP) targeting the formyltetrahydrofolate synthetase (FTHFS) gene, which is used as a marker gene for acetogens using the Wood-Ljungdahl pathway (21). The biocatalytic activities of the electrodes were compared using electrochemical techniques and analysis of the dissolved and gaseous reaction products.

## RESULTS

### Overall current production in both MECs.

**FIG 1:**
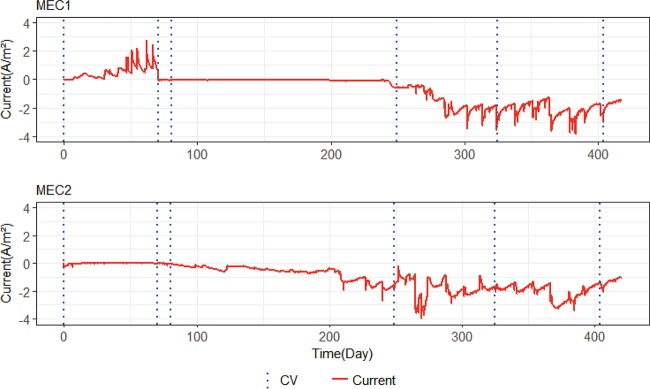
Current generation with time in MEC1 and MEC2. The positive current in MEC1 represents the current when the anodes were controlled at −0.2 V versus SHE. The negative current in both MECs represents the current when cathodes were controlled at −0.65 V versus SHE. Dashed lines indicate when normal operation was stopped and CV tests were carried out.

**FIG 2:**
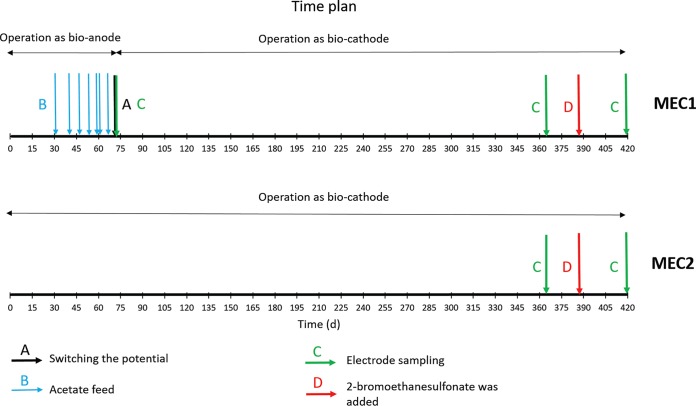
Schematic time plan for MECs. Arrows represent different actions that took place during the experiment.

Figure 1 shows the total current produced by MEC1 and MEC2. In MEC1, at the potential of −0.2 V versus SHE, the electrodes were working as anodes, and a positive current was generated. The negative current in both MECs represents the current produced when the electrodes were controlled at −0.65 V versus SHE and they were working as cathodes. The increase in current in MEC1 after 6 days shows the biological activity on the surface of the anodes. Microorganisms began to oxidize acetate and deliver electrons to the electrodes. Drops in current in MEC1 occurred when acetate was consumed in the nutrient medium. When this occurred, the medium was replaced (Fig. 2). The current reached around 2.5 A/m^2^ before the potential was switched to −0.65 v versus SHE in MEC1. After lowering the potential, the current dropped to 0.016 ± 0.007 A/m^2^ for approximately the next 170 days. This showed that the microorganisms dominating on bioanodes were not capable of operating as biocathodes when the potential was switched. About 170 days after the potential switch, the cathodic current increased to around 0.6 A/m^2^, and for the rest of the experiment, it fluctuated between 0.6 A/m^2^ and 3.6 A/m^2^, which showed that biocathodes had been enriched.

In MEC2, the cathodic current was initially 0.0078 ± 0.0077 A/m^2^. Bioelectrochemical activity was observed to increase after approximately 83 days, when the cathodic current reached around 0.1 A/m^2^. For the next 120 days, the current increased to approximately 0.6 A/m^2^ and fluctuated between 0.6 A/m^2^ and 3.3 A/m^2^ until the end of the experiment. Occasional drops in current generation happened when the reference electrode malfunctioned. Another reason for temporary drops in current was the cyclic voltammetry (CV) tests, which can have an effect on the activity of the electrodes due to variation in the potential applied to the MECs during the tests (22).

### Individual assessment of electrodes.

**FIG 3:**
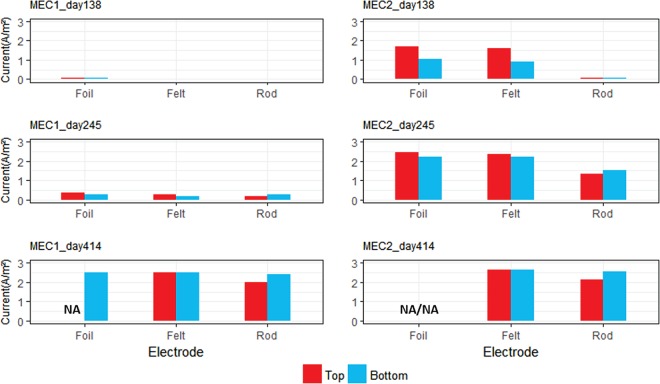
Disconnection tests for measuring current production by the individual electrodes in MEC1 and MEC2. Top, three electrodes that were installed from top of the MECs (graphite foil, carbon felt, and graphite rod from top); bottom, duplicates that were installed near the bottom.

Disconnection and CV tests were carried out to investigate the individual performance of each electrode in both MEC1 and MEC2. Disconnection tests were done by measuring the current generation before and after disconnecting one electrode from the potentiostat. The difference between total current generation before and after disconnecting the electrode showed the individual current production for that specific electrode. Three tests were done 138, 245, and 414 days after starting the MECs. Figure 3 shows the results of the disconnection tests from the different biocathode materials in MEC1 and MEC2. On days 138 and 245, the graphite foil and carbon felt electrodes produced more current than the graphite rod electrodes. However, on day 414 at the end of the study, all electrode materials produced a similar current. It should be noted that the current densities shown in Fig. 3 were calculated based on the projected surface areas of the electrodes. However, the carbon felt has a significantly higher actual surface area because it consists of a large number of intertwined carbon fibers.

**FIG 4:**
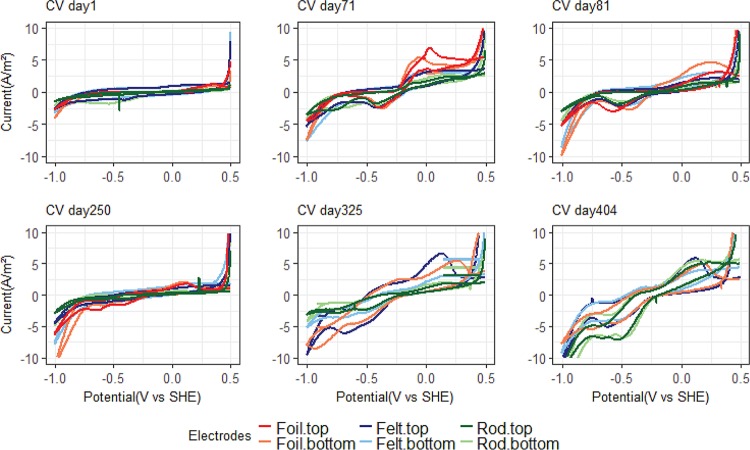
Six different CV tests for MEC1. The first two tests were done before switching the potential from −0.2 to −0.65 V versus SHE, and the other four tests were done after switching the potential. One graphite foil electrode was removed from MEC1 on day 313 due to technical problems.

The results from the CV tests for MEC1 are shown in Fig. 4. The first two tests were carried out before lowering the potential. The CV test after 71 days showed an increase in current at −0.2 V versus SHE. This type of anodic peak is usually seen in CV tests with acetate-fed bioanodes (e.g., 23) and indicates that acetate is being oxidized bioelectrochemically. The graphite foil electrode showed a higher and more distinct anodic peak than the other electrode materials. Directly after switching the potential, after 81 days of incubation, the CV tests still showed some catalytic waves. An anodic peak at around −0.2 V versus SHE indicated that the acetate-oxidizing biofilm still responded to the CV. There are also some cathodic peaks, and the onset of H_2_ evolution, at the potential of −1 V versus SHE, appears to be slightly shifted to a more positive potential than the CV tests from day 1. However, only a very low cathodic current (0.016 ± 0.007 A/m^2^) was observed when the electrodes were controlled at −0.65 V versus SHE (Fig. 1). From day 250 and onwards, the CV tests have a different shape, showing several reduction peaks. The onset of H_2_ evolution is markedly shifted from −1 V versus SHE to a more positive potential, especially on days 325 and 404. Reduction peaks were observed at potentials of −0.24 V, −0.4 V, and −0.6 V versus SHE. The final CV test, at 404 days after incubation, was carried out after inhibition of methanogens with 2-bromoethanesulfonate, and this showed that an acetogenic biofilm developed on rod electrodes, which produced significantly higher cathodic current at −0.5 V versus SHE than the methanogenic biofilm before inhibition.

**FIG 5:**
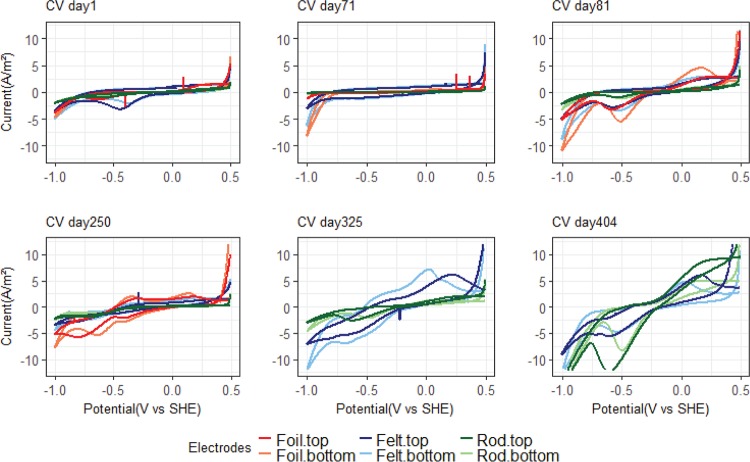
Six different CV tests for MEC2. Two graphite foil electrodes were removed from the MEC on day 271 due to technical problems.

Figure 5 shows six different CV tests for MEC2. On day 81, sustained cathodic current generation had begun when the cathode was controlled at −0.65 V versus SHE, and a distinct reduction peak is observed around that potential in the CV. As time progresses, the H_2_ evolution peak is more and more clearly shifted toward a more positive potential. Similar to MEC1, several peaks appear in the voltammograms, indicating the presence of several redox-active components with different redox potentials on the electrode surfaces. Except for day 404, the graphite felt and graphite foil electrodes had a steeper increase in cathodic current peaks, which corresponds to the higher current densities generated by these two electrode materials than with the graphite rod electrodes in MEC2 during the initial phase of the experiment (Fig. 3). The final CV tests, at day 404, showed a clear biological response despite the methanogens being inhibited.

### Methane and acetate production.

**FIG 6:**
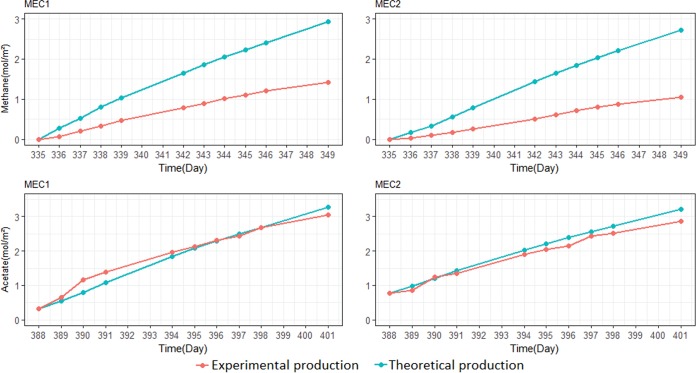
Methane (top) and acetate (bottom) production over a 2-week period in MEC1 and MEC2. The theoretical production refers to the methane or acetate that should be produced from the current which flowed through the MEC. Experimental production refers to the methane or acetate that was measured in the experiment.

During the experiment, methane was produced in the cathode chamber as the only noticeable gas. Figure 6 shows the results for the period between day 335 and day 349. The methane production is compared with the theoretical methane production.

The methane accumulation in MEC1 was approximately 0.1 mol/m^2^ · day. The methane concentration was around 50% of the theoretical methane calculated based on the average current flow during the 2-week period. The methane production in MEC2 was approximately 0.075 mol/m^2^ · day, which was approximately 38% of the theoretical production. The methane production in MEC1 was higher than in MEC2 due to the higher current generated in MEC1. However, in both MECs, the methane production was less than the theoretical value, which can be explained by systematic gas losses either from the reactors or during sampling and analysis. Microbial growth can also partly explain the discrepancy between the theoretical and measured values. However, methanogens are only expected to use 8% of the electrons for assimilation (24); thus, gas loss was likely the major reason. Furthermore, total organic carbon (TOC) and high-performance liquid chromatography (HPLC) analysis showed there was no dissolved organic carbon produced during the 2-week period, which confirms that systematic gas losses occurred in the reactors. Similar methane production rates were measured for both MECs in other 2-week periods (see Fig. S3 in the supplemental material).

Figure 6 also shows the acetate production for both MECs between day 388 and day 401 after the addition of 10 mM 2-bromoethanesulfonate to the nutrient medium. MEC1 and MEC2 produced acetate at a rate of 0.218 mol/m^2^ · day (0.82 mM/day) and 0.204 mol/m^2^ · day (0.61 mM/day), respectively. In both MECs, the acetate production curve had a slope similar to that of the theoretical acetate production curve. This showed that almost 100% of the current was converted to acetate in both cells; thus, the losses through the membrane were negligible. Theoretical acetate production was calculated based on current generation in the reactors. The acetate production rate was slightly higher in MEC1 than in MEC2 due to the slightly higher current that was produced during the 2-week period. Hydrogen gas was produced up to a maximum of 0.26 mmol and 0.04 mmol in MEC1 and MEC2, respectively, after 4 days, when the inhibitor was added for the first time. Then, the concentration of hydrogen decreased to nondetectable levels after 5 days.

### Microbial community analysis.

**FIG 7:**
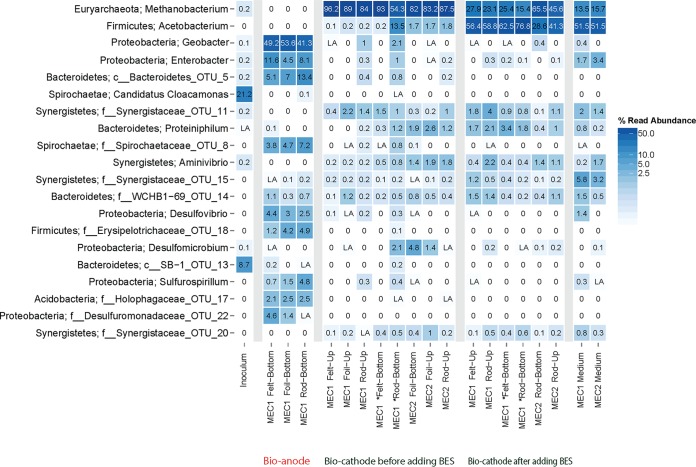
Relative abundances of the 20 most abundant 16S rRNA gene sequences in the bioanodes, biocathodes before and after adding 2-bromoethanesulfunate (BES), medium at the end of the experiment, and inoculum. LA refers to abundances of <0.1%. *, electrodes that were placed in MEC1 after removing bioanodes.

Figure 7 shows the relative abundances of the 20 most abundant operational taxonomic units (OTUs) in the MECs, based on 16S rRNA gene sequences. The inoculum contained a diverse community of bacteria distributed among the phyla Spirochaetes, Bacteroidetes, and Proteobacteria. The community on the bioanodes in MEC1, after 71 days of operation, had shifted considerably to a community dominated by Geobacter sp. (>40%) on all electrode materials. On the biocathodes, after 363 days of operation, the most abundant sequences were affiliated with the genus Methanobacterium (phylum Euryarchaeota), with a relative abundance over 50% in both MEC1 and MEC2, before adding the methanogen inhibitor 2-bromoethanesulfonate. Even after 30 days of 2-bromoethanesulfonate treatment, Methanobacterium was the second largest OTU. After 2-bromoethanesulfonate addition, Acetobacterium sp. (phylum Firmicutes) was the most abundant group of bacteria, with over 40% relative abundance. Apart from the dominating OTUs on the bioanodes and biocathodes, many bacterial phyla were present at a lower relative abundance (less than 5%). Also, these less-abundant OTUs were different in abundance over time. Ordination analysis showed that the inoculum, anodes, and cathodes formed three distinct clusters. The communities on the cathodes before and after the addition of 2-bromoethanesulfonate were also somewhat separated from each other (Fig. S1).

TRFLP analysis targeting the FTHFS gene showed distinct shifts in the microbial community similar to those for the whole bacterial community in a comparison of the bioanodes and the biocathodes before and after the addition of 2-bromoethanesulfonate. Out of 12 different TFRs, 5 TFRs (132, 232, 328, 448, and 528 bp) were exclusive for the bioanodes, and the remaining 7 TFRs (80, 108, 128, 212, 226, and 404 bp) were exclusive for the biocathode. Two TRFs (128 and 212 bp) only occurred on the biocathodes after the addition of 2-bromoethanesulfonate. The TRFs 632 and 636 bp (which represent the uncut fragment) were observed in all sample groups (Fig. S2).

## DISCUSSION

### Successions in microbial community composition.

The hypothesis at the start of this study was that the enrichment of bioanodes in MEC1 would facilitate the start-up of biocathodes. However, when the potential was switched from −0.2 V to −0.65 V versus SHE in MEC1, only a very low cathodic current was observed for about 170 days. This was despite the fact that in MEC1, when the electrodes were initially operated as bioanodes fed with acetate, the microbial community was dominated by Geobacter sp. at a relative abundance of >40%. Both Geobacter spp. and Desulfovibrio spp., which were also abundant on the bioanodes, have been shown previously to catalyze hydrogen production on biocathodes. However, previous studies with these microorganisms used cathode potentials from −0.7 to −1 V versus SHE, which are lower than the −0.65 V versus SHE used in our study (9, 25). The difference in results could possibly also be explained by differences at the species level, e.g., different species of Geobacter and Desulfovibrio were enriched in other studies compared to the present study. This might explain why the bioanodes did not generate cathodic current.

On an anode, Geobacter sp. can generate electricity by transferring electrons directly to a solid electrode, in the absence of an electron shuttle (26), via pili (27, 28). Previous studies have shown that Geobacter spp. are highly selected for in acetate-fed bioanodes (29–32). Other electrogenic microorganisms detected in our anode biofilms included Desulfovibrio sp. (2.5 to 4.4%) and potentially bacteria within Desulfuromonadaceae (0 to 4.8%) and Sulfurospirillum (0.7 to 4.8%). Desulfovibrio spp. are often found on bioanodes (33), and previous research has shown that pure cultures of Desulfovibrio desulfuricans can generate current in microbial fuel cells by direct electron transfer (34). Desulfuromonas spp. are also known to transfer electrons to anode surfaces (26), and Sulfurospirillum spp. have been observed at high abundances on biocathodes, where they may be involved in electron transfer (10).

Acetogens were expected to play a key role on the biocathodes, as they are capable of reducing CO_2_, and several acetogens have previously been shown to be electrochemically active (35). TRFLP of the key acetogen gene FTHFS showed a distinct shift when the electrodes were converted from anodes to cathodes. The five TRFs observed on the bioanodes were completely absent on the biocathodes. Instead, distinct cathode biofilm communities developed that were highly similar in MEC1 and MEC2 (Fig. S1 and S2), indicating that the selective forces shaping the specific cathode communities were in fact profound, given the different paths taken for the two MECs, from the founding seed sludge community to mature biocathodes. At present, these TRFs are difficult to identify, as no database exists exclusively for the FTHFS gene. In the seed sludge, only a fraction (<0.3%) were archaea, and virtually no methanogens were detected, despite a sequencing depth exceeding 2,000 reads per sample. Despite this, OTUs identified as Methanobacterium spp., which are known as hydrogenotrophic methanogens (36), were dominating the biocathodes at relative abundances of 54 to 97% in MEC1 and MEC2, and CH_4_ was identified as the only generated product in both reactors. In many MECs, Methanobacterium spp. and Methanobrevibacter spp. dominate the microbial communities (13, 37–39). Previously, Cheng et al. (13) showed that both a pure culture of Methanobacterium palustre and a mixed culture dominated by that archaeon could produce methane by reducing carbon dioxide, using a cathode as electron donor. Other studies have also found that Methanobacterium spp. and mixed cultures of hydrogenotrophic methanogens can produce methane either through direct electron transfer from the cathode or indirectly via hydrogen (37, 39, 40). Compared to the archaeal communities, the function of the bacterial communities on the biocathode is not as straightforward. However, just as for the methanogenic archaea, specific bacteria different from those in the inoculum and the anode (MEC1) proliferated on the cathode in both MECs. Even though the role of these bacteria is not clear, they most likely have multiple roles for the overall function and therefore the methane production on the cathodes, as recently indicated (38). For example, less-abundant members of microbial communities may be important for shuttling electrons from the cathode surface to the methanogens, e.g., by catalyzing H_2_ generation.

The biocathodes in both MECs generated current densities varying between 0.6 A/m^2^ and 3.6 A/m^2^. This is comparable to the results of a previous study by van Eerten-Jansen et al. (41), which reported average current densities of about 2.9 A/m^2^ generated by methane-producing graphite felt biocathodes enriched at a potential of −0.7 V versus SHE. Although the current densities in MEC1 and MEC2 eventually reached similar levels, the biocathodes enriched from bare electrodes at −0.65V versus SHE in MEC2 started up significantly faster than the biocathodes preenriched as bioanodes in MEC1. The reason for this could be that the specific microbial community enriched on the bioanodes in this study was not capable of cathodic current generation at −0.65 V versus SHE, and a complete transformation of the microbial community on the electrode surfaces was needed before current generation could commence. This is in contrast to some previous studies which have shown that preenrichment of bioanodes facilitated biocathode start-up (7, 15, 42).

When 2-bromoethanesulfonate was added to inhibit methanogens, the Methanobacterium spp. decreased in relative abundance, and Acetobacterium sp. increased in relative abundance from 0.1 to 13.5%, before inhibitor addition, to 29 to 77% at 32 days after the addition. Other studies of biocathodes operated with 2-bromoethanesulfonate as an inhibitor have also found Acetobacterium spp. in the microbial communities (10, 43, 44). For example, Su et al. (43) found that the biocathode community was dominated by a relative of Acetobacterium woodii. Nevin et al. (35) tested the bioelectrochemical activity of several acetogenic bacteria, including three Sporomusa spp., two Clostridium spp., and Moorella thermoacetica, and showed that these groups were capable of accepting electrons from a cathode and produce organic acids. Interestingly, Acetobacterium woodii was found to be unable to directly use electrons from the cathode surface (11, 35). Acetobacterium spp. are, however, known to produce acetate by oxidizing hydrogen and reducing carbon dioxide (45). Two new FTHFS gene TRFs (128 and 212 bp) emerged after methanogens were inhibited in the reactors. *In silico* digestion of the FTHFS gene of Acetobacterium woodii (accession no. NC_016894.1) showed a fragment different from those obtained in the experiment TRFLP. Thus, the OTU enriched on the biocathodes after the addition of 2-bromoethanesulfonate likely did not represent this species but rather other Acetobacterium spp. or other acetogens.

### Mechanisms of CH_4_ and acetate production.

Electron transfer from the cathode surface to the microorganisms producing methane and acetate could theoretically take place either via direct transfer to the microbial cell or via an intermediate, such as hydrogen and acetate. The results from this study suggest that hydrogen was involved as an intermediate. The dominating microorganisms under both CH_4_-producing (Methanobacterium spp.) and acetate-producing (Acetobacterium spp.) conditions are known hydrogenotrophs. When the methanogen inhibitor was added to the reactors, there was no noticeable change in current generation. If the Methanobacterium spp. had directly accepted electrons from the cathode surface, inhibiting its activity should have resulted in lower current production before a new electrochemically active species had established on the cathode surface. However, since no reduction in current occurred, the Methanobacterium spp. most likely produced CH_4_ via an intermediate, such as H_2_. In both MECs, the increase in current at around −1 V versus SHE (Fig. 4 and 5) corresponds to the hydrogen production reaction. The current peak at −1 V versus SHE increased over time and was shifted to more positive potentials, also observed previously by Marshall et al. (46), which suggests that the biofilms on the surfaces of the cathodes could catalyze hydrogen production. However, except for low levels of hydrogen detected during the first 4 days after addition of the methanogen inhibitor, hydrogen was generally not detected in the headspace of the reactors. This suggests that any hydrogen produced was rapidly consumed by other microorganisms in the reactors. The detection of some H_2_ gas after methanogen inhibition supports the hypothesis that H_2_ was acting as a mediator in the system. In fact, it has recently been shown that methanogenic consumption of dissolved H_2_ at the cathode can prevent H_2_ accumulation in the headspace (47). Then, the question arises, what was catalyzing the H_2_ production? Bare carbon and graphite electrodes cannot generate the current densities observed in this study at a potential of −0.65 V versus SHE (Fig. 1). Therefore, H_2_ production must have been biologically catalyzed in the reactors. Hydrogenases are reversible enzymes that can catalyze both the oxidation and production of H_2_. Free enzymes present in the culture medium (48), whole cells containing hydrogenases (25, 49), and even dead cells and cell debris (50) can catalyze H_2_ production on cathodes. For example, Deutzmann and Spormann (49) showed that a coculture of Acetobacterium woodii and the strain IS4, which catalyzed H_2_ production on the cathode, could generate acetate at a rate of 0.14 to 0.18 mol/m^2^ · day at −0.5 V versus SHE. van Eerten-Jansen et al. (41) also concluded that in biocathodes operated at −0.7 to −0.9 V versus SHE, CH_4_ production was mainly taking place through intermediate production of H_2_ and acetate. Given the high specificity of the cathodic biofilm community and the inability of the anodic biofilm in MEC1 to assist in the start-up of biocathodes, one may speculate that the microbes assisting in the generation of hydrogen were in fact specific, although it remains unknown which community members provided the activity.

The CV tests showed that the catalysis on the biocathodes improved over time. Under normal operation conditions, the biocathodes were operated at −0.65 V versus SHE. However, in the last CV tests, cathodic current started to increase at −0.24 V versus SHE in both MECs (Fig. 5). This suggests that it may be possible to increase the operation potential of the biocathodes and thereby decrease the input energy required to run the system. Indeed, Pisciotta et al. (15) operated biocathodes at a potential of −0.439 V versus SHE. The last CV was carried out on day 404 when methanogens were inhibited and acetogens dominated the electrodes. This CV showed the strongest biological response, with a large reduction peak at about −0.5 V versus SHE. The change in the CV on this occasion compared to the previous tests could be because of the change in microbial composition when the methanogens were inhibited and acetogens took over.

### Electrode material.

Three electrode materials, graphite foil, carbon felt, and graphite rods, were tested in this study. All materials had the same projected surface areas in the reactors. Graphite foil has a very smooth surface, so the actual surface area is probably similar to the projected area. Graphite rods have a rougher surface, and carbon felt consists of a large number of intertwined carbon fibers, so the actual surface area is much larger than the projected area. Despite this difference in actual surface area, all electrodes generated similar levels of current in the end of the study. We speculate that the reason for this could be that biofilm covering the outer part of carbon felt limited the diffusion of chemical components to and from the inner surfaces of the felt. Thus, the higher actual surface area did not automatically translate into higher current. During start-up of the reactors, the graphite foil and the carbon felt produced current faster than the graphite rods. The CV scans from day 1 of the experiment (Fig. 4 and 5) showed a somewhat lower cathodic current at the negative scan limit (−1.0 V versus SHE) for graphite rods than the other two electrode materials. This cathodic current is associated with the hydrogen evolution reaction. When the electrodes were polarized at −0.65 V versus SHE, low levels of abiotic H_2_ production at the graphite foil and carbon felt electrodes could have facilitated a more rapid establishment of hydrogen-oxidizing biofilms on these electrodes. Ordination (Fig. S1) showed a small difference in microbial communities between different electrode materials. Generally, biocathodes with the same material from the same type of electrode clustered together, e.g., graphite rods in MEC1 and graphite foil in MEC2 (Fig. S1). This suggests that even though the most abundant members of the microbial communities were the same among the cathodes, differences associated with the electrode material still existed.

## MATERIALS AND METHODS

### Bioelectrochemical cell.

Two Plexiglas double-chamber MECs were operated as batch reactors, with a total volume of 750 ml for each chamber (34 by 6 by 3.5 cm). Six different electrodes, with 2 graphite foils (catalog no. 43083, 1 mm thick; Alfa Aesar), 2 carbon felts (catalog no. 43199, 3.18 mm thick; Alfa Aesar), and 2 graphite rods (catalog no. 14738, 6.15 mm diameter; Alfa Aesar), with a projected area of approximately 11.4 cm^2^ each, were installed in one working chamber of each MEC. One graphite foil, one carbon felt, and one graphite rod electrode were installed at the top half of each MEC, and another set of electrodes was installed at the bottom half. Three graphite rod electrodes were used as counterelectrodes in the other chamber of each MEC. The MECs also contained an Ag/AgCl reference electrode, with an offset of 0.197 V versus the standard hydrogen electrode (SHE), located in the working chamber. A nutrient medium with a total volume of 750 ml was circulated through each working chamber at a flow rate of 50 ml · min^−1^. The two chambers in each MEC (working and counter) were separated by a cation exchange membrane (CMI-7000; Membranes International, Inc.).

### Inoculum and nutrient medium.

The MECs were inoculated with 20 ml of a mixture of raw municipal wastewater and anaerobic digester sludge at a ratio of 9:1 and then filled to 750 ml with a nutrient medium containing, per liter, 0.1 g of KCl, 0.6 g of KH_2_PO_4_, 0.25 g of NH_4_Cl, 3 g of NaHCO_3_, 0.1 g of MgCl, and 0.03 g of CaCl. A trace element and vitamin solution, as described by Marshall et al. (46), was added (20 ml per liter) to the medium mixture. Cysteine (0.5 g per liter) was also added to the nutrient medium as an oxygen scavenger. The medium was replaced every week during bioanode enrichment and every 2 to 4 weeks when cathodic current started to be generated. During the replacement process, the medium was sparged with Ar-CO_2_ gas (85%/15%) to prevent aerobic conditions. Sodium acetate (1.62 g per liter) was added to MEC1 during the first 71 days to enrich bioanodes. The counterelectrode chamber in both MECs contained the same nutrient medium without the trace element and vitamin solution.

### Reactor operation.

The two MECs were operated with two different start-up strategies. (i) The electrodes in MEC1 were enriched as acetate-oxidizing bioanodes by controlling the potential at −0.2 V versus SHE for the first 71 days. Then, acetate was removed from the nutrient medium, and the potential was decreased to −0.65 V versus SHE, which made the electrodes work as cathodes during the rest of the experiment. (ii) MEC2 was operated by controlling the cathode potential at −0.65 V versus SHE from the start of the experiment. Three different electrodes in MEC1 were harvested for microbial community analysis before switching the potential. To replace these three electrodes, three new electrodes were added to MEC1. To investigate the ability of the microorganisms to produce hydrogen, acetate, or other volatile fatty acids (VFA), 10 mM 2-bromoethanesulfonate was added to the nutrient medium after 387 days in order to inhibit any methanogens present on the biocathodes (Fig. 2). At day 363, before adding 2-bromoethanesulfonate, a part of the cathode electrodes from both MECs was cut and stored in a freezer for microbial analysis. Cathode samples were also cut and stored in a freezer for microbial analysis at the end of the experiment.

### Analytical methods.

During MEC operation, the potential was controlled using Wenking MLab potentiostats, and the current was recorded by the MlabSci470c sequencer multichannel potentiostat software (version 4.7.0). VFA concentrations were analyzed using a high-performance liquid chromatography (HPLC) system equipped with a UV detector (Shimadzu) and an Aminex HPX-87H column (Bio-Rad), with 5 mM H_2_SO_4_ eluent pumping at 0.5 ml/min. Biogas was collected in gas bags installed at the top of the cathode chambers and was analyzed by gas chromatography (Micro GC system; Agilent). The production rate was measured for several 2-week periods during the experiment. At the start of each such 2-week period, the liquid in the reactor was replaced by fresh medium, and the gas bags were filled with 500 ml of argon-CO_2_ gas. Theoretical methane was calculated for both MECs over these 2-week measurements. Theoretical methane is defined as moles of methane per projected area of the cathode surface, which can be produced theoretically considering the current flow in the MECs (equation 1).

(1) $$\text{C}{\text{O}}_{2}+8{\text{H}}^{+}+8{\text{e}}^{-}\to \text{C}{\text{H}}_{4}+2{\text{H}}_{2}\text{O}$$

The pH was measured by a pH sensor (WTW Multi 350i). The bioelectrochemical activity of the electrodes was investigated using cyclic voltammetry (CV). CV was done with scan limits of 0.5 V and −1.0 V versus SHE at a scan rate of 5 mV/s. All electrochemical tests were carried out with the Wenking MLab potentiostat. The current generation of each electrode was investigated by disconnecting the electrode from the potentiostat and measuring the drop in the current after disconnection. The cathode potential was controlled at −0.65 V versus SHE during the disconnection test.

### Microbial community analysis.

In order to analyze the microbial communities present on the surface of the electrodes, samples were collected before lowering the potential, before adding methanogen inhibitor, and at the end of the experiment (Fig. 2). DNA was extracted using the FastDNA spin kit for soil (MP Biomedicals). DNA concentrations were measured using a NanoDrop ND-1000 spectrophotometer (Thermo Scientific). DNA extracts were diluted to 10 ng/μl with sterile water. The 16S rRNA genes were amplified in duplicate using forward primer 515′F (GTGBCAGCMGCCGCGGTAA) and reverse primer 806R (GGACTACHVGGGTWTCTAAT) to amplify the V4 region sequences of the bacterial and archaeal 16S rRNA genes (20). Dual-index labeling for primers was done according to the approach described by Kozich et al. (51). Duplicate PCRs were carried out in a 20-μl volume using 1 μl of target DNA, 17 μl of AccuPrime *Pfx* SuperMix (Life Technologies), and 1 μl of the forward and reverse primers, respectively. The PCR was conducted using a Bio-Rad T100 thermal cycler with a program consisting of activation (95°C, 5 min); 30 cycles of denaturation (95°C, 20 s), annealing (50°C, 20 s), and elongation (68°C, 60 s); and a final elongation (68°C, 10 min). The products were purified (Agencourt AMPure system; Beckman Coulter), normalized per concentration, and pooled prior to sequencing on an Illumina MiSeq system using the MiSeq reagent kit version 2. The sequences obtained were processed using the UPARSE workflow (52). Merged sequences with expected errors exceeding 3 or a sequence length below 200 bp or above 300 bp were discarded for further analysis. OTU clustering (97% similarity) with chimera filtering was done using the cluster_otus command. A total of 189,122 read sequences were obtained for all samples, and these clustered into 788 OTU. Subsampling to 2,000 sequences per sample was done before analysis with R using the ampvis package (53).

The FTHFS gene was amplified with previously developed primers (21), with some modifications (54). For the restriction reactions, the amplified FTHFS gene fragment of ∼635 bp was purified using the E-Gel Safe Imager system and E-Gel SizeSelect 2% agarose gels (Invitrogen). Ten nanograms of DNA was digested overnight with the restriction enzyme AluI (New England BioLabs), and the TRFLP analysis protocol was as described by Müller et al. (21).

### Accession number(s).

Raw data files were deposited to the National Center for Biotechnology Information (NCBI) Sequence Read Archive (SRA) database available online, with study accession no. PRJNA412029 (SRP118808).

## Supplementary Material

Supplemental material
