# Supplementary material for: Effect of Start-Up Strategies and Electrode Materials on Carbon Dioxide Reduction on Biocathodes
Source: Appl Environ Microbiol. 2018 Jan 31;84(4):e02242-17. doi: 10.1128/AEM.02242-17 (PMC5795077; doi:10.1128/AEM.02242-17)
Supplement: Supplemental material [file supp_84_4_e02242-17__index.html]

Supplemental material 

# Effect of Start-Up Strategies and Electrode Materials on Carbon Dioxide Reduction on Biocathodes

## Supplemental material

- Supplemental file 1 -

  NMDS plot for microbial communities on different electrode materials (Fig. S1); table based on TRFLP data (Fig. S2); methane and acetate production (Fig. S3).

  PDF, 883K
