# Supplementary material for: Effect of Start-Up Strategies and Electrode Materials on Carbon Dioxide Reduction on Biocathodes
Source: Appl Environ Microbiol. 2018 Jan 31;84(4):e02242-17. doi: 10.1128/AEM.02242-17 (PMC5795077; doi:10.1128/AEM.02242-17)
Supplement: Supplemental material [file AEM.02242-17_zam004188331s1.pdf]

Supplemental Material

Effect of start-up strategies and electrode materials on carbon dioxide reduction on bio-cathodes

Soroush Saheb-Alam,[a#] Abhijeet Singh,[b] Malte Hermansson,[c] Frank Persson,[a] Anna Schnürer,[b] Britt-Marie Wilén, [a] Oskar Modin[a]

Chalmers University of Technology, Department of Architecture and Civil Engineering, Division of Water Environment Technology, Gothenburg, Sweden[a]; Swedish University of Agricultural Sciences, Department of Molecular Sciences, BioGas group, Unit of Microbiology, Uppsala, Sweden[b];University of Gothenburg, Chemistry and Molecular Biology, Gothenburg, Sweden[c]
#Address correspondence to Soroush Saheb-Alam, soroush.sahebalam@chalmers.se.

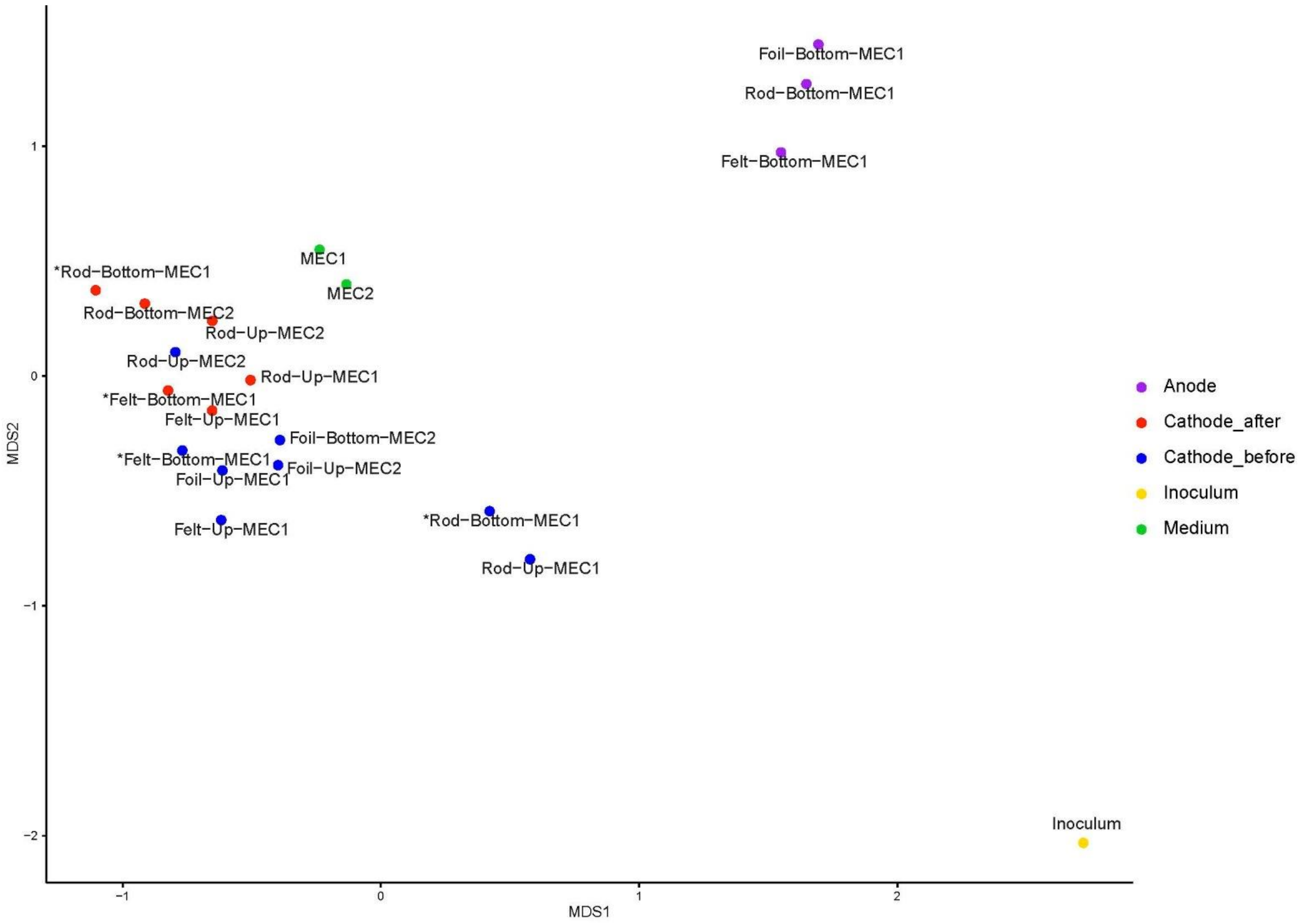

Figure S1. NMDS plot for microbial communities on different electrode materials before and after adding BES, medium and inoculum. *Electrodes that were placed in MEC1 after removing bio-anodes.

	Only anodes					Only cathodes before		Only cathodes after		Only cathodes			All groups	
	TRF (bp)													
**Sample**	**132**	**232**	**328**	**448**	**528**	**80**	**404**	**128**	**212**	**108**	**208**	**266**	**632**	**636**
Anode-(MEC1-Felt-Bottom)	29%		29%											42%
Anode-(MEC1-Foil-Bottom)	9.7%	8.7%	4.1%	24%										17%
Anode-(MEC1-Rod-Bottom)				13%	12%								16%	59%
Cathode-before-(MEC1-Foil-Up)										13%	16%	18%	53%	
Cathode-before-(MEC2-Foil-Bottom)											28%			72%
Cathode-before-(MEC2-Foil-Up)						19%	16%							65%
Cathode-after-(MEC1-Rod-Up)									2.8%	16%	17%	24%	40%	
Cathode-after-(*MEC1-Rod-Bottom)									14%		18%	18%	50%	
Cathode-after-(MEC2-Rod-Bottom)									29%		24%	7.9%	19%	21%
Cathode-after-(MEC2-Rod-Up)								6.8%	23%	8.3%	24%	12%	11%	16%

Figure S2. Table based on TRFLP data. The relative abundances of the different TRFs are shown. “Before” and “after” refer to before and after 2-bromoethanesulfonate addition. Asterisk (*) means that the electrode was placed in MEC1 after removing some bio-anodes for sampling.

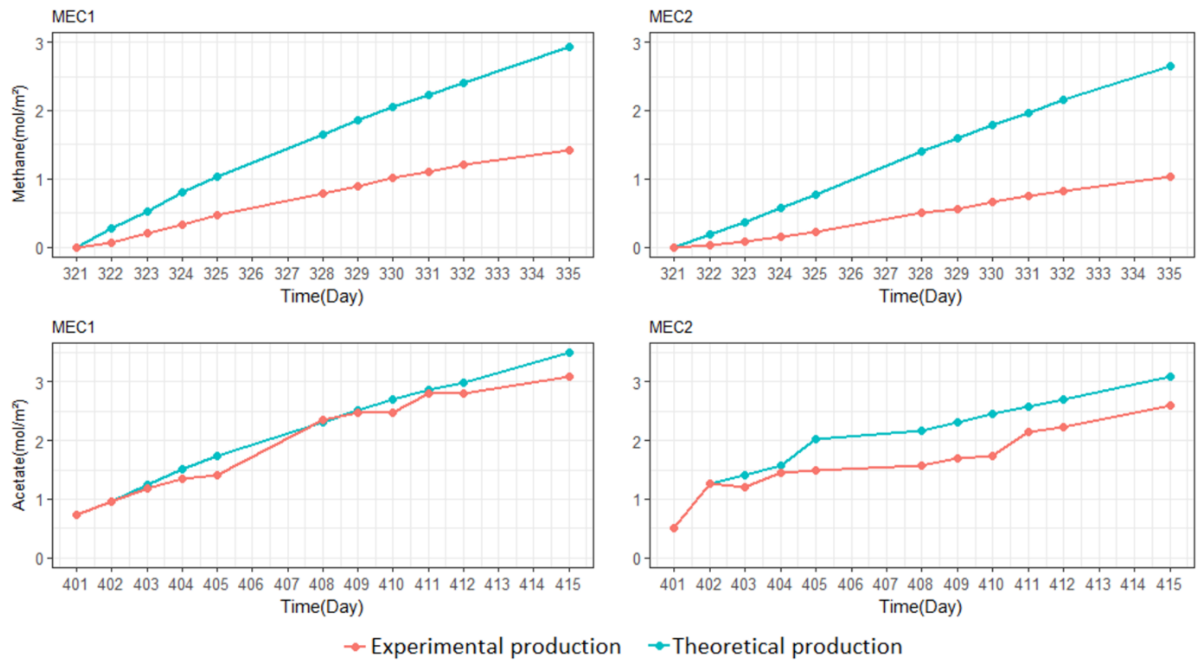

Figure S3. Methane and acetate production over two-week periods in MEC1 and MEC2. The theoretical production refers to the methane/acetate that should be produced from the current which flowed through the MEC. Experimental production refers to the methane/acetate that was measured in the experiment.
